# A Context-Sensing Mobile Phone App (Q Sense) for Smoking Cessation: A Mixed-Methods Study

**DOI:** 10.2196/mhealth.5787

**Published:** 2016-09-16

**Authors:** Felix Naughton, Sarah Hopewell, Neal Lathia, Rik Schalbroeck, Chloë Brown, Cecilia Mascolo, Andy McEwen, Stephen Sutton

**Affiliations:** ^1^ Behavioural Science Group Department of Public Health and Primary Care University of Cambridge Cambridge United Kingdom; ^2^ Computer Laboratory University of Cambridge Cambridge United Kingdom; ^3^ Health Behaviour Research Centre University College London London United Kingdom; ^4^ National Centre for Smoking Cessation and Training London United Kingdom

**Keywords:** mobile phone app, smoking cessation, context sensing, tailoring, geofence, just-in-time adaptive intervention, JITAI, ecological momentary intervention, craving

## Abstract

**Background:**

A major cause of lapse and relapse to smoking during a quit attempt is craving triggered by cues from a smoker's immediate environment. To help smokers address these cue-induced cravings when attempting to quit, we have developed a context-aware smoking cessation app, Q Sense, which uses a smoking episode-reporting system combined with location sensing and geofencing to tailor support content and trigger support delivery in real time.

**Objective:**

We sought to (1) assess smokers’ compliance with reporting their smoking in real time and identify reasons for noncompliance, (2) assess the app's accuracy in identifying user-specific high-risk locations for smoking, (3) explore the feasibility and user perspective of geofence-triggered support, and (4) identify any technological issues or privacy concerns.

**Methods:**

An explanatory sequential mixed-methods design was used, where data collected by the app informed semistructured interviews. Participants were smokers who owned an Android mobile phone and were willing to set a quit date within one month (N=15). App data included smoking reports with context information and geolocation, end-of-day (EoD) surveys of smoking beliefs and behavior, support message ratings, and app interaction data. Interviews were undertaken and analyzed thematically (N=13). Quantitative and qualitative data were analyzed separately and findings presented sequentially.

**Results:**

Out of 15 participants, 3 (20%) discontinued use of the app prematurely. Pre-quit date, the mean number of smoking reports received was 37.8 (SD 21.2) per participant, or 2.0 (SD 2.2) per day per participant. EoD surveys indicated that participants underreported smoking on at least 56.2% of days. Geolocation was collected in 97.0% of smoking reports with a mean accuracy of 31.6 (SD 16.8) meters. A total of 5 out of 9 (56%) eligible participants received geofence-triggered support. Interaction data indicated that 50.0% (137/274) of geofence-triggered message notifications were tapped within 30 minutes of being generated, resulting in delivery of a support message, and 78.2% (158/202) of delivered messages were rated by participants. Qualitative findings identified multiple reasons for noncompliance in reporting smoking, most notably due to environmental constraints and forgetting. Participants verified the app’s identification of their smoking locations, were largely positive about the value of geofence-triggered support, and had no privacy concerns about the data collected by the app.

**Conclusions:**

User-initiated self-report is feasible for training a cessation app about an individual’s smoking behavior, although underreporting is likely. Geofencing was a reliable and accurate method of identifying smoking locations, and geofence-triggered support was regarded positively by participants.

## Introduction

Between one-third and one-half of smokers in high-income countries make a quit attempt every year [1-3]. However, over half of those attempting to quit relapse within 1 month [2]. A major reason for this high relapse rate is a failure to manage cravings brought about by smoking cues from the environment. Cue-induced cravings are implicated in almost half of all smoking lapses [4], and early lapses to smoking are highly predictive of subsequent relapse [5-7], including when experimentally induced [8]. Cues are often dichotomized into proximal cues (eg, a lighter), those directly associated with smoking behavior that may be common to multiple smoking settings, and distal cues (eg, a smoker’s kitchen), which are present during smoking but not directly linked to it [9]. Both cue types have been found to trigger cravings to smoke in experiments [9]. While cue-induced cravings are not alleviated by the most commonly used cessation medications [10,11], the timely use of cognitive or behavioral coping strategies can help smokers manage or avoid these craving episodes without lapsing [10,12]. However, among smokers who use any strategies to prevent lapse, the ones most commonly used appear to be the least evidence based [13,14]. Given that tobacco smoking is the second-highest contributing factor to the global burden of disease [15], finding interventions to help smokers better address cue-induced cravings is a high priority.

Mobile phone-based ecological momentary interventions (EMIs) have potential to deliver cognitive-behavioral lapse-prevention support to help smokers address cue-induced cravings. However, user-triggered lapse-prevention support within EMIs, such as texting a *HELP* or *CRAVE* keyword to a short message service (SMS) cessation support system, is generally found to be used infrequently [16,17], as are apps relying on users to initiate access to support content, based on initial evaluations [18]. System-triggered lapse-prevention support driven by fixed schedules [19], random timing [20], or a combination of the two [16] are likely to be limited in their ability to deliver *just-in-time* lapse support, as found in a recent study showing no difference in lapse-prevention strategy use between smokers who did and did not receive an EMI [14].

A third approach, a hybrid of user- and system-triggered support, is context-triggered lapse-prevention support. Mobile phone sensors can continuously and unobtrusively interpret the context of a situation and trigger support if the individual is deemed to be in a high-risk situation for a lapse, often termed *just-in-time adaptive interventions* (JITAIs). Outside of the smoking cessation field, context sensing has been used to train an app to predict mood states [21] and tailor system-triggered behavioral advice for a physical activity and eating behavior app [22]. While the concept of context-aware smoking cessation interventions has been discussed [23,24], we are not aware of any research investigating EMIs that deliver cessation support that is context triggered in real time. Given the likely growth in use of context sensing in health apps, insights into the feasibility and user perspective of this approach are of high importance, especially in light of perceived barriers highlighted by young adults when discussing hypothetical tracking and context-sensing health behavior apps [25]. These barriers include expectations for the collection of accurate and detailed data without burdensome or boring data input; concerns about counterproductive effects if triggered support cues, rather than stops, behavior; and data privacy concerns. These remain important and largely unexplored issues.

We have developed a context-aware smoking cessation mobile phone app, *Q Sense*, that, in addition to more common features, delivers behavioral support triggered by and tailored to an individual’s real-time context to prevent smoking lapses. In advance of a smoker’s nominated quit date, the app employs a user-initiated real-time smoking episode and context-logging system that collects location data from the sensors in the mobile phone using open-source software libraries [26]. Once a smoker’s quit date has passed, the app passively monitors their location and when they enter or dwell within a geofence [27]—a system-generated virtual perimeter—it triggers support messages, tailored to the context information it has collected for that location.

A context-aware EMI such as Q Sense requires both user and sensor data to train the system to infer specific contexts. Ecological momentary assessment (EMA) studies using mobile phones have found that the compliance of user-initiated reports of smoking are somewhat lower compared with when smokers are given prompts [28,29]. These studies have shown that just over half of all smoking episodes are reported when relying on users to initiate reporting [28,29]. However, a limitation with these studies in the context of real-world usage is their experimental nature; mobile phones, financial incentives, and compliance training were provided, and compliance was assessed over less than one week [28,29]. Furthermore, these studies provide little insight into why smokers underreport when recording smoking in real time.

To support its development as a smoking cessation tool, we sought to investigate the barriers and facilitators of user engagement with Q Sense and learn about its support delivery system under natural conditions without external reinforcement. To gain a user-centered perspective, we adopted a mixed-methods design. By integrating quantitative and qualitative components, mixed-methods designs can provide greater insight into phenomena than either method alone [30,31], and can minimize the limitations of each type of data [32].

There were four main objectives, each with explicit quantitative and qualitative elements [33]:

1. Assess smokers’ engagement with self-initiated reporting of smoking (quantitative) and identify reasons for any noncompliance (qualitative).

2. Assess the app’s location-sensing (quantitative) and perceived (qualitative) accuracy in identifying user-specific high-risk locations for smoking.

3. Explore the feasibility of the geofence trigger mechanism (quantitative) and smokers’ views on this, and how the support it delivers could be optimized (qualitative).

4. Identify any technological limitations (quantitative), problems, or privacy concerns (qualitative) in everyday use.

## Methods

### Design and Participants

**Figure 1:**
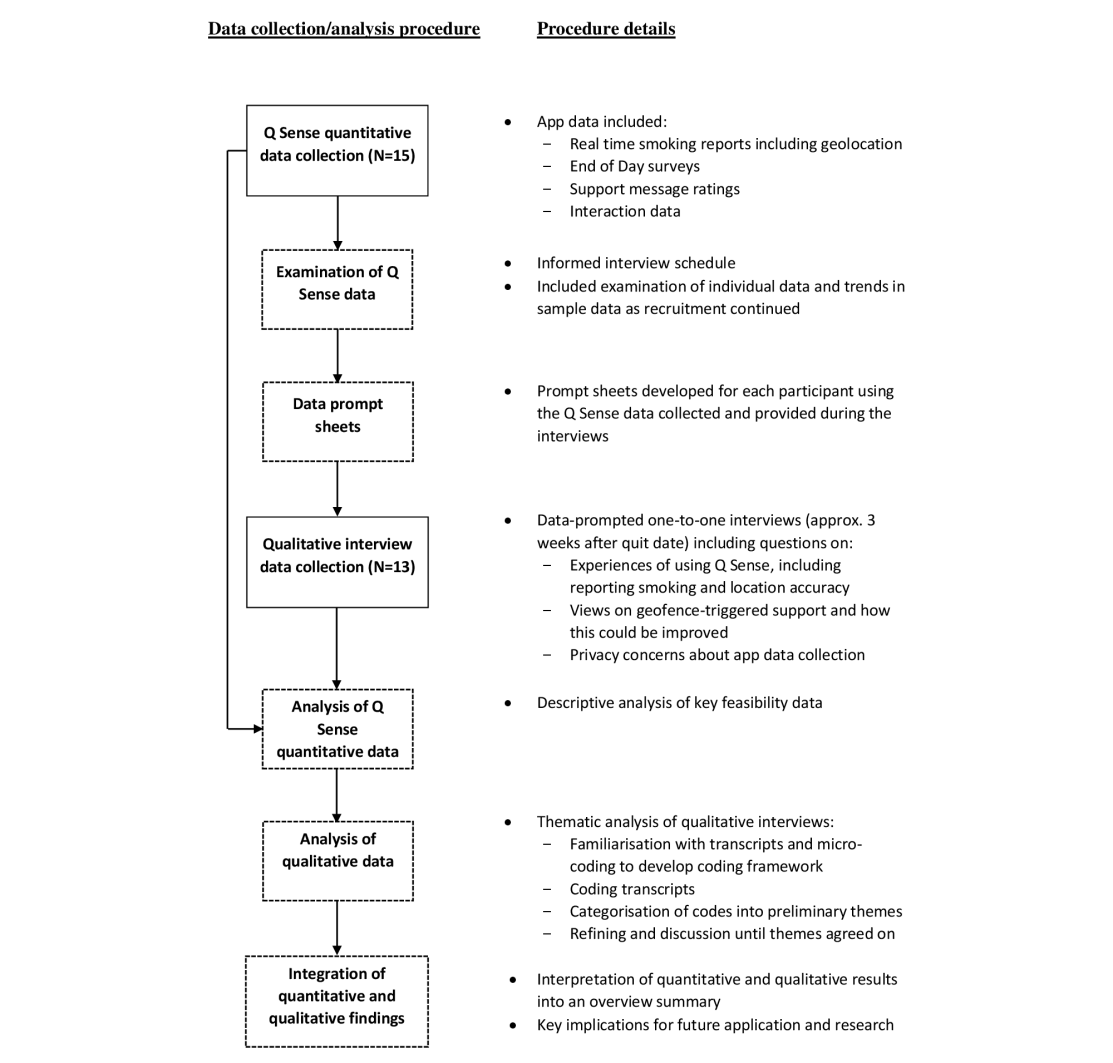
Procedural diagram of the explanatory sequential mixed-methods design used.

An explanatory, sequential, mixed-methods design was used [33]. Guided by this design, a preliminary examination of quantitative data collected by participants using the Q Sense app informed data-prompted, qualitative, one-to-one, semistructured interviews [34]. The interviews aimed to help explain the quantitative findings and generate insight into app usage behavior. The two strands were therefore connected or “mixed” during data collection. Analysis of data was undertaken separately and sequentially, and integrated during interpretation. Figure 1 provides a procedural diagram of the design.

Participants were recruited using convenience sampling. Eligibility criteria included the following: being a current tobacco smoker, aged 16-70 years, interested in quitting smoking, willing to set a quit date within a month but not less than one week, primary use of an Android mobile phone, and willing to participate in a one-to-one interview. Electronic and paper advertisements were placed on social media websites, university staff and student mailing lists, company communication pages, newspapers, and local shops and leisure centers. The research team emailed those expressing an interest on the study website with a participant information sheet before getting in touch by telephone to answer any questions and take verbal consent.

### Procedure

Consenting participants were sent a link by email and SMS text message to install Q Sense. Participants were asked to use Q Sense for the duration of the pre-quit-date period and for at least 2 weeks post-quit date. When installing Q Sense, the app requests permission to collect users' locations.

Q Sense (version 1) implements a smoking cessation EMI as a three-stage process, with support informed by two theory-guided SMS text message cessation interventions [16,35], learning theory [36], and taxonomy of smoking behavior change techniques [37].

In the first stage, *Prepare and Learn*, users set a quit date after completing an 11-item demographic and smoking survey. They were then asked to log every time they smoked tobacco in the time leading up to their quit date by opening the app, tapping an *I'm smoking* button, and completing a short assessment. Each self-assessment contained five questions about their current psychological and situational context just before they smoked: mood, stress, strength of urges to smoke (adapted from the Mood and Physical Symptoms Scale [38]), current situation (*Home*, *Working*, *Socializing*, or *Other*), and the presence of others (*I am alone*, *Friends/Family*, *Colleagues*, or *Others*) and whether the others were smoking. If the smoker reported smoking more than four times in the same proximity, the device created a geofence (ie, virtual perimeter) around that area [27]. A geofence is a circular area around a location defined by a latitude and longitude, in this case determined by the location of the smoking reports, and a radius that was set at 100 meters. Android Location Services, which uses multiple location sensors including the global positioning system (GPS), informs the app when the device enters or dwells in—defined by Q Sense as 3 hours or more—the geofence.

The combined self-assessment and location data were also sent to a server, which responded by sending a tailored support or feedback message. The support message was selected from a prepopulated database that matches the user's 11-item demographics and smoking survey, and the feedback message was based on the statistical patterns in the user's smoking reports (eg, “Did you know? Based on 12 reports, 25% of the times you smoke you are working.”).

The app's second stage, *Commit to Quit*, begins when the user's quit date has arrived and lasts for 28 days. In this stage, when the app detected that a user had entered a geofence and stayed in that location for at least 5 minutes, a support message notification was automatically triggered. Further support message notifications were then triggered after each 3-hour interval of dwelling in the same geofenced location. Tapping on the notification delivered the support message. The content of the geofence-triggered support messages were tailored to the information collected from the smoker when they reported smoking in that specific location. For example, if a user had, on average, reported moderately high stress levels when smoking at home and then entered a home geofence, the server would sometimes return a support message written for the home context and for someone experiencing moderate-to-high stress. The mean length of these messages was 198 characters (SD 50). If users reported smoking during this stage, they received postreport lapse and relapse prevention support messages (mean length 219 characters, SD 45).

During the first two stages, users also received a morning notification linking to a tailored quitting preparation message or smoking fact, during the *Prepare and Learn* stage, and support addressing outcome expectancies of stopping smoking or general encouragement, during the *Commit to Quit* stage (mean length 181 characters, SD 63). After any support message was viewed, users were invited to rate the message using a 5-star rating scale. Users were also able to view a summary of their recorded smoking episode data split by situation as part of their app *profile*. Every day, users were also invited to complete an *end-of-day* (EoD) survey that recorded number of cigarettes smoked that day (categorical), strength and frequency of urges to smoke [38], and abstinence self-efficacy. The app also passively tracked users’ location by collecting samples from their devices’ location sensors in the background every 15 minutes.

In the third stage, *Maintain the Change*, the app was in a passive state and did not deliver any proactive support messages, though the profile and reporting features with postreporting lapse and relapse prevention support messages were still active. See Multimedia Appendices 1-6 for app screenshots.

After participants had either used Q Sense for approximately 2 weeks post-quit date, or appeared to have disengaged (ie, no use for a week), they were invited to participate in a face-to-face or telephone interview. Interviews were audiotaped and lasted approximately 30-60 minutes. App data for each participant was examined prior to each interview and used to inform the interview schedule. To assist in generating discussion and participant views [34], each participant was provided with a printed summary of their app data, including a map of their smoking locations (see Multimedia Appendix 7). General patterns emerging from the app data as participants completed the study informed the focus of the discussion in subsequent interviews. Interview questions focused on participants’ experiences of using the app and, in particular, reporting smoking and the accuracy of smoking location identification, views on the geofence-triggered support and how this could be improved, and privacy concerns about app data collection. All interviews were transcribed verbatim. Participants who were interviewed received a £10 shopping voucher.

### Feasibility Measures

Feasibility is defined as the assessment of whether processes and procedures are possible and practical to do easily or conveniently. App-based feasibility measures included the following: mean frequency and location of smoking reports, current location (ie, background samples), proportion of EoD surveys completed, proportion of days where EoD smoking category exceeded daily smoking reports, proportion of smoking reports with geolocation data, accuracy of geolocation generated by the device (68% confidence interval of true location), number of active geofences created per participant, proportion of participants receiving any geofence-triggered support messages, the number of geofence support messages delivered, the time to view geofence support messages, and the proportion of support messages rated.

### Analysis

In line with the explanatory, sequential, mixed-methods design, the two connected but different strands of data were analyzed separately and the findings presented sequentially [33]. The findings from both strands were combined at an interpretative level to generate key conclusions.

Descriptive statistics of feasibility measures were generated using SPSS version 22 (IBM Corp) and Microsoft Excel. Thematic analysis was used to analyze the interview transcripts, informed by Braun and Clarke’s phases of thematic analysis [39]. Two researchers (SH, RS) read and familiarized themselves with the transcripts and then microcoded several to identify initial concepts and categories as part of developing a preliminary coding framework. The two researchers then both coded three interview transcripts using NVivo 10 (QSR International) to check the coherence of the coding framework and refine it where discrepancies were identified. Then all remaining interviews were coded by both researchers, and the coded content was merged. Coded content was then read through, and codes were categorized into areas of primary focus, informed by the research questions. Preliminary themes and subthemes were identified from these categories and were refined after their fit with the raw and coded data had been reviewed. After discussions between two researchers (SH, FN), agreement on the primary themes was reached.

## Results

### Participants

A total of 41 people expressed an interest in participation; 21 met the inclusion criteria of whom 15 participated (71%). Exclusions were due to not having access to an Android mobile phone (11/41, 27%) or wanting to quit in less than one week (9/41, 22%). Of the 15 participants, there were 8 males (53%) and 7 females (47%), and most reported smoking between 6 and 10 cigarettes per day. Of the 15 participants, 13 (87%) were interviewed either face-to-face (9/13, 69%) or by telephone (4/13, 31%). All participants set a quit date, with 5 out of 15 (33%) reporting planning to use an e-cigarette to support their attempt. See Table 1 for participant characteristics. Out of 15 participants, 3 (20%) stopped using the app during or at the end of the pre-quit-date phase due to disengagement (1/3, 33%) or technical problems (2/3, 67%).

**Table 1:** Participant characteristics (N=15).

Characteristic		n (%) or mean (SD)
Gender (female), n (%)		7 (47)
**Age in years, n (%)**		
	18-24	2 (13)
	25-34	9 (60)
	35-44	2 (13)
	45+	2 (13)
**Cigarettes per day, n (%)**		
	1-5	2 (13)
	6-10	8 (53)
	11-15	5 (33)
	16-20	0 (0)
	21+	0 (0)
Smoked first cigarette within 30 minutes, n (%)		6 (40)
Number of days from enrollment to quit date, mean (SD)		20 (5)
Lives with other smokers, n (%)		4 (27)
Planning to use an e-cigarette to help in quit attempt, n (%)		5 (33)

### Quantitative Findings

Table 2 provides a summary of app-collected feasibility outcomes. The mean pre-quit-date period was 19.8 (SD 4.8) days. During this time, the mean number of smoking reports per participant logged on Q Sense was 37.8 (SD 21.2), or 2.0 (SD 2.2) per day, reported at home (246/491, 50.1%), work (152/491, 31.0%), while socializing (30/491, 6.1%), and in *other* situations (63/491, 12.8%). Background location samples were broadly in line with the locations where participants smoked in terms of time spent in that location: home (60.0%), work (15.0%), and *other* locations (25.0%). Out of 12 participants, 6 (50%) self-reported smoking after their quit date, with a mean of 3.7 (SD 1.9) reports, and a mean number of days until first lapse of 9.0 (SD 5.7). Of these reports, 68.2% were at work, 27.3% were at home, and 4.5% were at *other* locations. The median time taken to complete a smoking report was 12.9 seconds (interquartile range [IQR] 7.9; mean 17.5, SD 36.8).

**Table 2:** Feasibility outcomes for use of the Q Sense app (N=15).

Outcome		Mean (SD) or n (%)
Number of smoking reports per participant pre-quit date, mean (SD)		37.8 (21.2)
**Reported situations of smoking reports (pre-quit date) (total reports N=491), n (%)**		
	Home	246 (50.1)
	Working	152 (31.0)
	Socializing	30 (6.1)
	Other	63 (12.8)
Proportion of days in pre-quit-date period where the number of smoking reports was lower than end-of-day (EoD) smoking category^a^ (total EoD surveys N=281), n (%)		158 (56.2)
Location capture for smoking reports^a^ (total reports N=513), n (%)		498 (97.0)
Accuracy of location capture (meters)^a,b^, mean (SD)		31.6 (16.8)
Geofence *smoking locations* generated per participant^a^_,_ mean (SD)		1.5 (0.7)
Participants receiving at least one geofence support message (N=9)^c^, n (%)		5 (56)
Geofence messages delivered per participant, who received any geofence messages, mean (SD)		40.4 (35.0)
**Compliance with end-of-day surveys, n (%)**		
	Pre-quit date^a^ (expected total number of surveys if 100% compliance N=250)	150 (60.0)
	Post-quit date (28-day period)^d^ (expected total number of surveys if 100% compliance N=336)	131 (39.0)
Total support messages delivered, n		1109
Support messages rated (N=1109), n (%)		933 (84.13)

^a^N=13: excludes 2 participants who stopped using Q Sense during the pre-quit-date phase.

^b^68% confidence interval of true location—generated by Android operating system.

^c^N=9: excludes participants who did not report smoking a sufficient number of times to create a geofence.

^d^N=12: excludes 3 participants, 2 who stopped using Q Sense during the pre-quit-date phase and 1 who only used Q Sense in the pre-quit-date phase.

On average, the EoD surveys were completed on 60.0% of days pre-quit date and 39.0% of the 28-day post-quit-date period—52.0% when excluding 3 participants that did not complete any post-quit-date surveys. EoD survey smoking category responses indicated that participants underreported smoking in the pre-quit-date phase on at least 56.2% of days. The EoD survey data also suggested that participants reduced their smoking during the pre-quit stage relative to baseline; on 43.7% of days, participants smoked at least one smoking category less than they indicated at baseline and only smoked more than their baseline category on 7.0% of days.

Geolocation was collected in 97.0% of smoking reports with a mean accuracy of 31.6 (SD 16.8) meters. A mean of 1.5 (SD 0.7) geofences (>4 smoking reports in one location) were created per participant with 13 out of 15 participants (87%) having at least one geofence created. Of 9 participants eligible to receive geofence-triggered support—having at least one active geofence and having spent time in that area post-quit date—5 (56%) received at least one geofence-triggered support message. A total of 202 geofence-triggered messages (aggregated mean delivery rate per day of 3.0 [SD 0.8] per participant) were delivered by the server. Of these, 60.4% (122/202) were *entry* and 39.6% (80/202) were *dwell* geofence messages.

**Figure 2:**
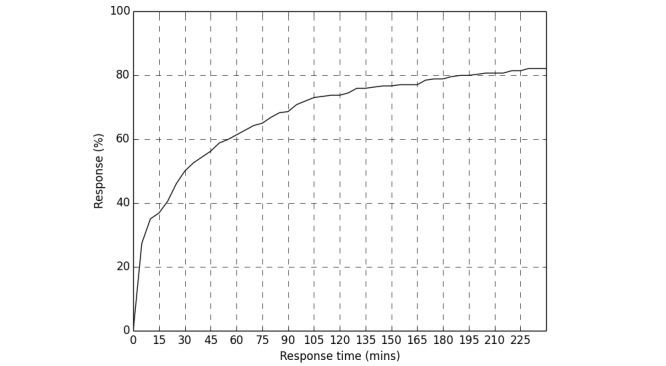
Cumulative distribution function of response times of opening app after geofence support notifications were generated.

A total of 1109 different support messages were delivered by the app (mean of 85.3 [SD 38.1] per participant) as a result of the participant tapping a message notification. Of these, 933 (84.13%) received a rating score from participants. For geofence-triggered support messages, 78.2% (158/202) of messages delivered were rated. App interaction data indicated that, of geofence-triggered support messages delivered, the median elapsed time between the generation of a notification and the user opening the app was 23.6 minutes (IQR 77.7). For all geofence-triggered support notifications, regardless of whether the notification was tapped on or not, on 50.0% (137/274) of occasions participants opened the app within 30 minutes of a notification being generated. Figure 2 shows the aggregated response pattern over time.

Regarding app activity, opening the app to report smoking, complete a survey, or view a support message represented 95.94% (7129/7431) of activity occasions, accessing the profile section represented 4.01% (298/7431) of occasions, and using the settings menu represented 0.54% (4/7431) of occasions.

### Qualitative Findings

#### Compliance With Reporting

Most participants stated that they had wanted to report smoking in real time, but struggled at times to do so. The main reason given was forgetting. Other reasons included not having the phone at hand, not wanting to appear rude around others, driving, not being in the mood, and not understanding the purpose of reporting at the time of smoking. Some participants viewed the EoD survey as an opportunity to make up for reports that they had missed during the day:

I started reporting each time I had a cigarette and I think that sort of petered off, but I know there will be an end of day and I can report it then.

Participant #20

Several participants stated that they reported less often the nearer they were to their quit date, particularly if they had set a date relatively far into the future:

Yes. It is like when you first start dieting, you do really well for the first week and then it sort of fades out doesn’t it?

Participant #9

Some participants described that the act of reporting smoking in itself led to them reduce their smoking frequency, as they became more self-aware and questioned their reasons for smoking:

When I was logging how much I am craving it was lower than what I thought it would have been without the app, which was really good. So that made me think, well, actually do I really need a cigarette now?

Participant #6

Several participants also stated that they were unaware that they could report smoking post-quit date. Where participants had reported post-quit date, they indicated that they had been generally happy to do so initially. However, 2 participants explained that they had stopped post-quit-date reporting after a few days of smoking, seemingly due to feelings of guilt or despondency:

The first couple of days I reported, then after that I chose not to. I don’t know why, I just didn’t. I probably felt like, oh God I haven’t quit, so I won’t use it kind of thing.

Participant #6

A number of suggestions were made to help increase reporting compliance. These included having programmable reminders; having the option to report smoking, including location, after the event, such as tapping on a map; having the option to complete shorter smoking reports; and receiving feedback during the day about the number of cigarettes smoked.

#### Locations of Smoking

Apart from a few rare exceptions, participants deemed the locations of their smoking reports as recorded by Q Sense as correct and accurate, based on the data prompt sheet superimposed maps. Participants were largely happy with the way the app used location sensing (eg, GPS and Wi-Fi), though a few made reference to areas with poor GPS and Wi-Fi signals, and 1 participant described feeling frustrated with the time it took for the app to identify his location on the map. Several participants also suggested that having more options available to describe their location setting (eg, *travelling*) would be helpful.

#### Geofence-Triggered Messages

Those receiving geofence messages were largely positive about their value, and described them as useful in providing distractions or alternatives to smoking:

It’s kind of good to have the reminder to say instead of having a cigarette take deep breaths and just take some time out to do some breathing exercise. It’s something that I know, but I wouldn’t think to do it, so it’s good to have that message.

Participant #20

One participant commented on the tailoring of a message when she was at home, and that she found this to be an important aspect in making it feel personalized:

But I guess it was kind of based on the time it knew I was at home or whatever, you know...those sort of trigger times it sort of sent a message to say, yes, so I felt it was aimed directly at me as opposed to just a random blanket message.

Participant #20

Feelings about message frequency were mixed, with one participant expressing that he received too many geofence-triggered messages and another that he would have preferred more. Three participants also explained that the geofence messages had on occasion reminded them about smoking, when they had not been thinking about it at the time:

But it just kept reminding me that I was smoking and I just wanted to forget about it, but it was kind of knowing when to get rid of the app.

Participant #8

When I see it pop up, I think Q Sense, I think “Oh fag” and that’s it, so it’s like you’re not even thinking about smoking and then it comes up and you’re thinking, “Oh I’ll have a fag now.”

Participant #16

However, these 3 participants did not specifically refer to the messages as triggering an urge to smoke and each suggested that the benefits of the messages outweighed the risks of being reminded:

Because even though the Champix tablets [meant] no cravings, I’m still thinking about smoking for some reason, but without a craving. And then when that message comes through it says like, “[Name], don’t do it.” I’m like, “Oh okay. Alright, I won’t!” [laughter]. I won’t do it [laughter]. I thought that was really helpful.

Participant #8

But yeah, some of those things, like the tips it sends you, they’re quite handy I think. It was quite useful to know really, the alternative options, because I’ve tried like the vapor pens and the e-liquids and stuff, they didn’t work for me.

Participant #16

In terms of message content, shorter messages, suggestions of alternatives to smoking, and messages that felt tailored to the situation were described as most helpful. Several participants also expressed a preference for more health- and risk-related geofence-triggered messages with harsher language to make risks more salient, and messages with novel information. Longer messages were seen as more time-consuming to read and, therefore, less useful.

Participants described being happy to rate messages received, and understood the rating system. Reasons for not reading or rating messages included being busy, not wanting reminders about smoking, and being unable to read them at work.

#### Technical Aspects and Data Privacy

Participants reported no noticeable impact of Q Sense on their phone’s battery life and, when asked, participants unanimously stated that they were unconcerned about privacy aspects of the data collected by the app. However, a few participants explained that their lack of concern was due to the study affiliation with a university and that their feelings would be different had the study been conducted by a commercial company:

I would have been happy to give more time, more personal data, things along those lines. Whilst if it was, I don’t know, [name of commercial pharmacy] or someone along those lines coming up with an app, I would have given them the bare minimum because I don’t trust where that data is going to go.

Participant #17

#### Other App Features and Optimization

Participants also discussed several more general features of Q Sense. Setting a quit date was often described as valuable for boosting commitment. Morning support messages were also described as being a helpful motivation boost, particularly once participants’ quit dates had arrived. In terms of future developments, suggestions included having the option to set a new quit date, enabling user preferences for the types of messages provided (eg, health information and motivational message), having cartoons or videos as well as text support, and including more graphics and visual displays. Several participants also suggested having a “human” element within the app, to link in with a support network or a stop-smoking advisor or service:

But it would definitely be useful to have contact, even if it was through messaging with a smoking cessation worker, because they do know more than most about smoking and how to quit.

Participant #22

## Discussion

### Principal Findings

This study provides novel and in-depth insights into the feasibility of a context-sensing smoking cessation app. A study strength is the connection of two types of data to address different dimensions of key study objectives. Overall, we found that collecting self-reported smoking behavior and geolocation data in real time via the app was feasible, and participants were engaged in this process. Participants often underreported their smoking behavior. While many of the barriers to reporting that were identified are potentially addressable through in-app intervention and improved explanation of the importance of real-time reporting, promoting app-based data entry remains a challenge [28,40]. Participants receiving support triggered by entering into, or dwelling within, a geofence demonstrated good message engagement and they were positive about its value.

Participants took on average less than 20 seconds to submit smoking data and were generally positive about the process, with the main barriers of real-time data entry connected to opportunity, forgetting, social inhibition, and misunderstanding the purpose, rather than boredom or burden [25]. The smoking reporting compliance rates observed were in line with prior experimental studies, which find smokers log just over half of what they smoke when self-initiated [28,41]. Providing prompts for reporting was one approach suggested by a number of participants to increase reporting compliance; providing the prompts themselves are unlikely to encourage or cue smoking. Another approach is to provide feedback on any mismatch between real-time reports made during the day and the total number of smoking episodes entered into the end-of-day survey. Where a mismatch occurs, this would include the provision of advice on the importance of real-time reporting and promoting techniques for increasing compliance. These approaches have been added into version 2 of Q Sense. However, low compliance may not represent a major barrier to training apps like Q Sense, providing the smoking reports logged represent an individual’s usual smoking behavior, which was broadly supported by the location-sampling data recorded in this study. This could lead to some situations being underrepresented, such as those where the individual may be inhibited to report smoking due to the presence of others.

Recording geolocation data for smoking episodes was feasible, reliable, and sufficiently accurate to justify the use of geofencing to identify high-risk areas for smokers. However, not all eligible participants received geofence-triggered support. An identified error in system logic resulted in at least two participants not receiving geofence-triggered messages. A further potential issue may have been the geofence radius, set at 100 meters. If location accuracy is poor and the estimated location is not fully inside the geofence perimeter, no geofence event occurs. Increasing the geofence radius may reduce this potential issue.

On average, participants each engaged with Q Sense to either complete reports or view a support message in more than 100 separate occasions over 6 weeks or less, and provided 74 support message ratings per participant. Those receiving geofence-triggered support had higher interaction occasions. While there is little published data on interaction with smoking cessation apps, evaluations to date have generally reported much lower frequency of interaction occasions, potentially due to reliance on proactive interaction from the smoker to access support content [18,42,43]. Our evaluation also enabled us to examine novel data on the time delay from geofence-triggered message notification to the opening of the app. Half of the geofence-triggered message notifications led to the message being viewed relatively promptly, within 30 minutes. However, many messages were viewed after a much longer delay. Future attention to time delay to response is important to identify the realistic limits of just-in-time adaptive interventions and to help inform the identification of opportunities when users are most receptive to engaging with support, such as after an episode of phone activity [44].

Concerns have been raised that EMI-delivered behavioral support could inadvertently prime individuals into thinking about engaging in the behavior they are aiming to avoid [24,25]. We found that while this concern was highlighted by a few participants, they reported being mindful of smoking much of the time anyway, supporting previous findings on this issue for an SMS text message-based cessation EMI [45]. In addition, these Q Sense participants regarded the benefits of a support message to outweigh the cons of being reminded about smoking. This is reinforced by evidence of a benefit of mobile phone-based EMIs for smoking cessation [46], and evidence showing that high-intensity prompts to record EMA data related to smoking and cravings reduce, rather than increase, cravings to smoke [47]. This recent EMA study also supports our unanticipated finding that the process of reporting smoking behavior was, for some, perceived to reduce their smoking rate, suggesting a potential self-monitoring benefit of EMA on smoking behavior [48].

Our findings highlight a number of approaches for optimizing apps like Q Sense. Suggestions from participants for overcoming reporting barriers centered around in-app reminders and feedback, as well as postevent and one-touch reporting features to combat forgetting and reporting constraints. Q Sense version 1 was designed for a relatively long pre-quit learning period to maximize the specificity of identifying locations where cue-induced cravings are likely to be experienced. However, smokers receiving specialist support have been found, on average, to select a quit date only 1 week into the future [16]. Furthermore, recent evidence indicates that the majority of smokers who download a cessation app will select the day of registration as their quit date rather than a future date (personal communication by Harveen Ubhi, March 15, 2016). To minimize the risk of damaging smokers' quitting motivation by insisting on long pre-quit-date training periods, apps relying on self-report data should be optimized to identify high-risk smoking locations in a short period. For Q Sense, this has subsequently been addressed in version 2 by lowering the frequency threshold of smoking reports to create a geofence, and enabling new geofences to be created around the locations where smokers report lapses. In addition, version 2 creates geofences for any smoking lapses reported during the quit attempt. An alternative approach as partially suggested by participants, which is relevant to smokers who might have a very short or absent pre-quit learning phase, would be to enable smokers to identify their smoking locations retrospectively, using address details or an interactive map, for geofence creation. This remains an area for exploration.

In addition to refinements described above, we have also added the following features to version 2 of Q Sense: an “inbox,” where all delivered support messages are stored; a navigable “library” of support messages, including the facility for users to write their own geofence support messages; a money-saving calculator in the profile section; and the facility to reset a quit date with prompts to do so after a specified number of reported lapses.

### Limitations

The technical issues preventing some participants from receiving geofence-triggered support reduced the information we were able to obtain on this intervention approach. This limits the conclusions that can be drawn about the feasibility of delivering this type of support, and the engagement that participants displayed with this support. While we discussed this type of support with all participants, future work is required to explore the user perspective and impact of context-triggered behavioral support. While we did not provide training on the use of Q Sense, in order to maximize ecological validity, inviting participants to be interviewed may have affected their app use and smoking behavior compared with having no direct contact with the study team. Future studies without the requirement for interview feedback might reduce this potential influence on participants. As a more general limitation, only Android users were eligible to participate in this study. Evidence has emerged of differences between smokers using Android and iOS phones to access a smoking cessation app; iOS users were more likely to make a serious quit attempt and set their quit date as the day of registration (personal communication by Harveen Ubhi, March 15, 2016). Therefore, we may have missed some important perspectives. Furthermore, as a consequence of seeking out an in-depth user-centered perspective, and our largely opportunistic sampling strategy, our sample may not be representative of the population of smokers who use mobile phones. In particular, our participants had low rates of daily smoking relative to other app-based smoking studies [18]. Future research with larger and more representative samples will enable a more precise estimate of usage, acceptability, and impact of context-triggered cessation support.

Future work is also likely to include the use of additional sensors to advance the identification of smoking behavior and high-risk contexts. The use of additional sensors would expand the scope for directly identifying proximal or other smoking cues that are present across settings, as identification would not be limited to geolocation; for example, the presence of other smokers using Bluetooth colocation traces [49,50].

### Conclusions

User-initiated self-report represents a largely feasible way to train a smoking cessation app about an individual's smoking behavior. While participants often underreported their smoking, the barriers to reporting smoking in real time were primarily related to environmental constraints and forgetting, rather than low motivation to report. Geofencing was a feasible and accurate method of identifying previous smoking locations representing high-risk situations during a quit attempt. Among those who experienced geofence-triggered support, the support was valued and there was high engagement with the delivered messages, although not all were viewed rapidly in response to an alert notification. Future work is required to expand our knowledge on the impact of context-tailored support, and how JITAIs can be optimized.

## Appendix

Multimedia Appendix 1 Q Sense screenshot—homepage.

Multimedia Appendix 2 Q Sense screenshot—reporting smoking.

Multimedia Appendix 3 Q Sense screenshot—my profile.

Multimedia Appendix 4 Q Sense screenshot—geofence-triggered support message.

Multimedia Appendix 5 Q Sense screenshot—daily support message.

Multimedia Appendix 6 Q Sense screenshot—lapse prevention support message.

Multimedia Appendix 7 Example of a data prompt sheet.

## Abbreviations

EMA: ecological momentary assessment
EMI: ecological momentary intervention
EoD: end of day
GPS: global positioning system
IQR: interquartile range
JITAI: just-in-time adaptive intervention
MRC: Medical Research Council
PHIND: Public Health Intervention Development
SMS: short message service
