# Supplementary figures and images for: A Context-Sensing Mobile Phone App (Q Sense) for Smoking Cessation: A Mixed-Methods Study
Source: JMIR Mhealth Uhealth. 2016 Sep 16;4(3):e106. doi: 10.2196/mhealth.5787 (PMC5045522; doi:10.2196/mhealth.5787)

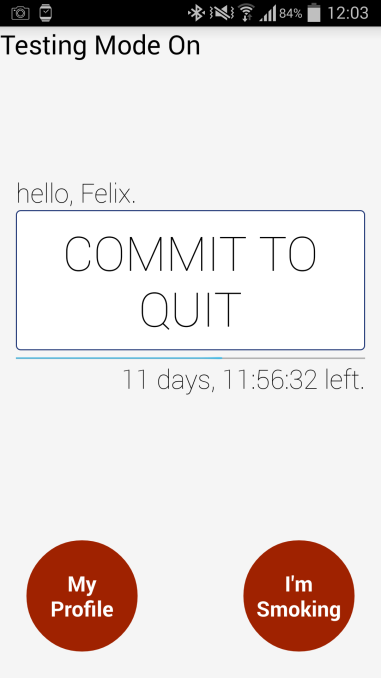

Supplement: Multimedia Appendix 1 [file mhealth_v4i3e106_app1.png]

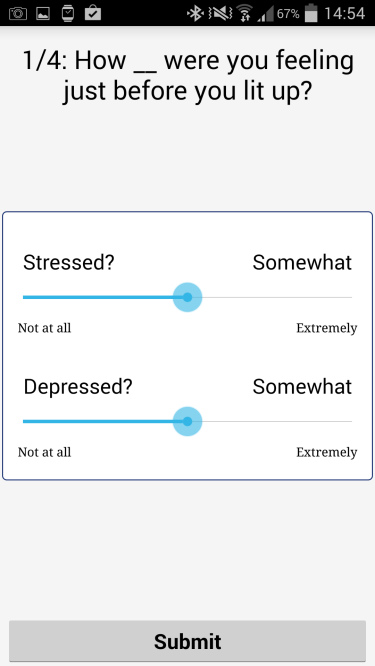

Supplement: Multimedia Appendix 2 [file mhealth_v4i3e106_app2.png]

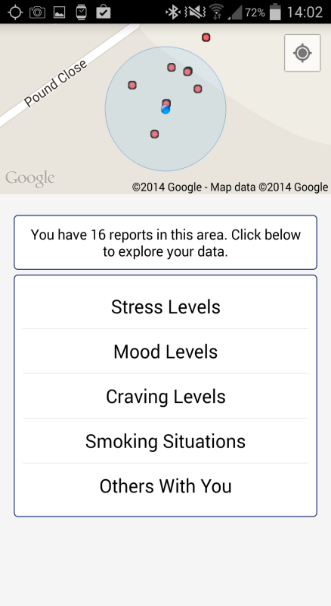

Supplement: Multimedia Appendix 3 [file mhealth_v4i3e106_app3.png]

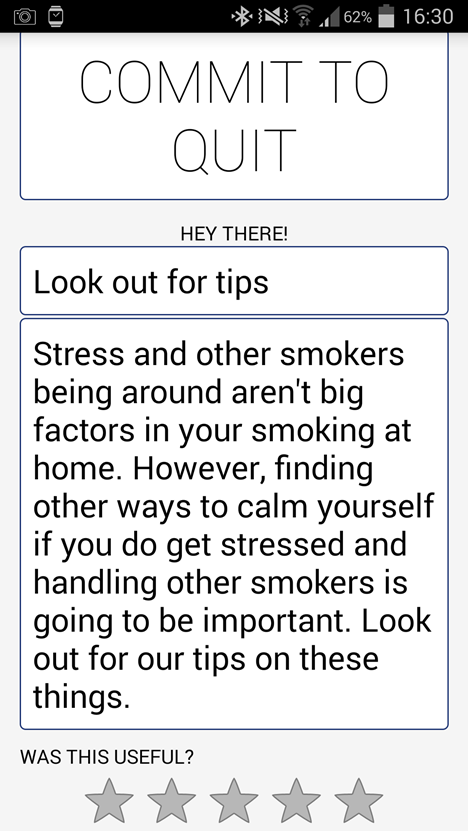

Supplement: Multimedia Appendix 4 [file mhealth_v4i3e106_app4.png]

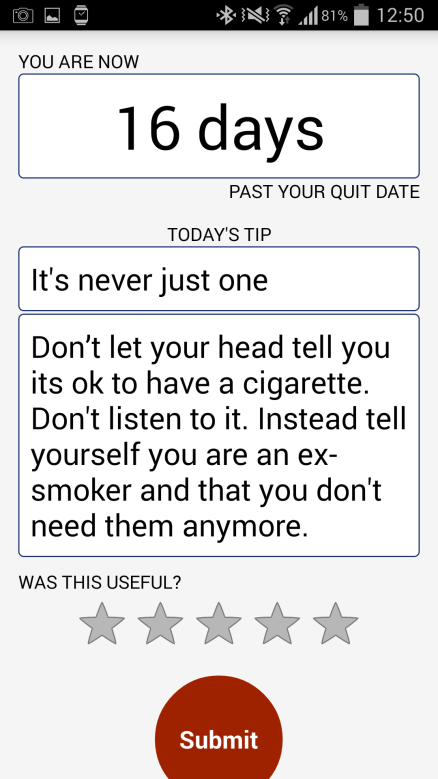

Supplement: Multimedia Appendix 5 [file mhealth_v4i3e106_app5.png]

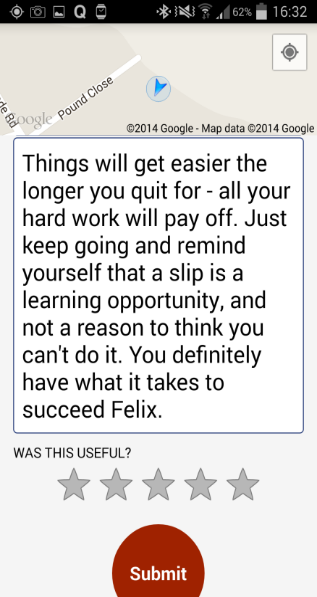

Supplement: Multimedia Appendix 6 [file mhealth_v4i3e106_app6.png]
