# Supplementary material for: A Context-Sensing Mobile Phone App (Q Sense) for Smoking Cessation: A Mixed-Methods Study
Source: JMIR Mhealth Uhealth. 2016 Sep 16;4(3):e106. doi: 10.2196/mhealth.5787 (PMC5045522; doi:10.2196/mhealth.5787)

average craving/stress levels throught the day

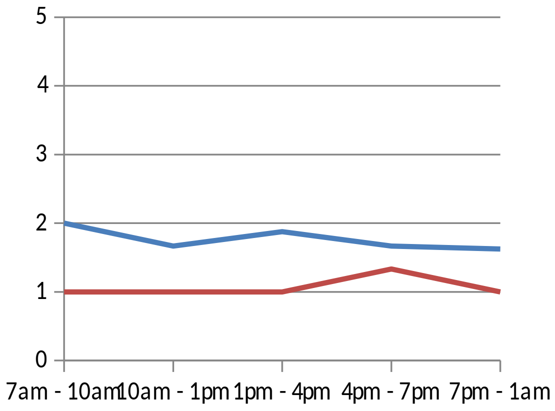

Total reports at different times of day

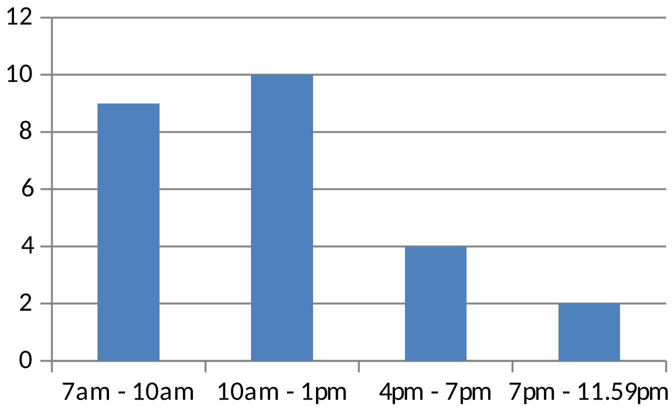

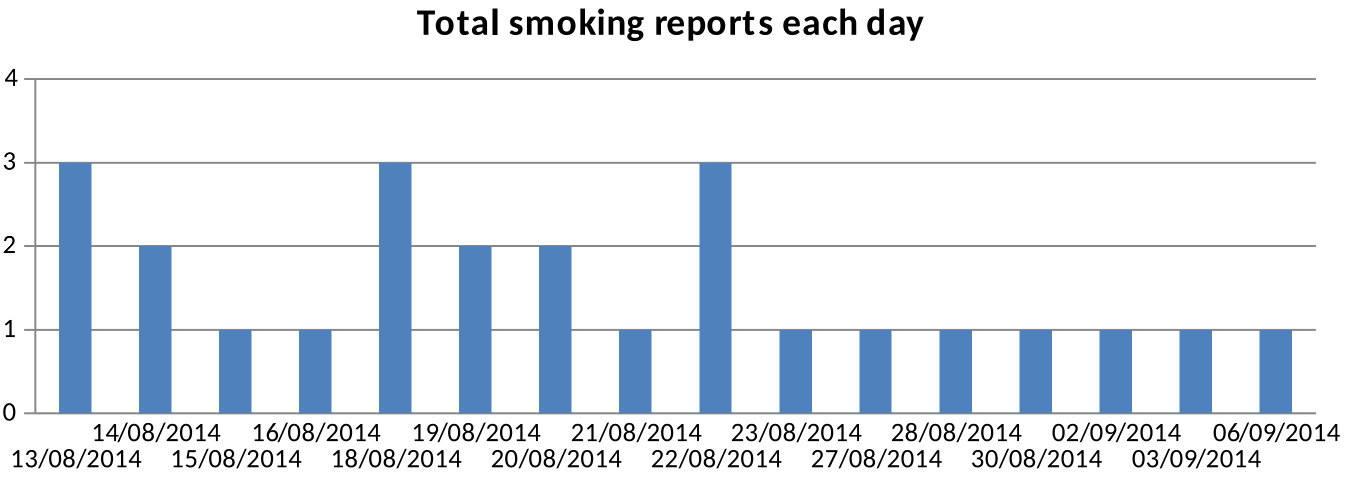

Smoking report locations:

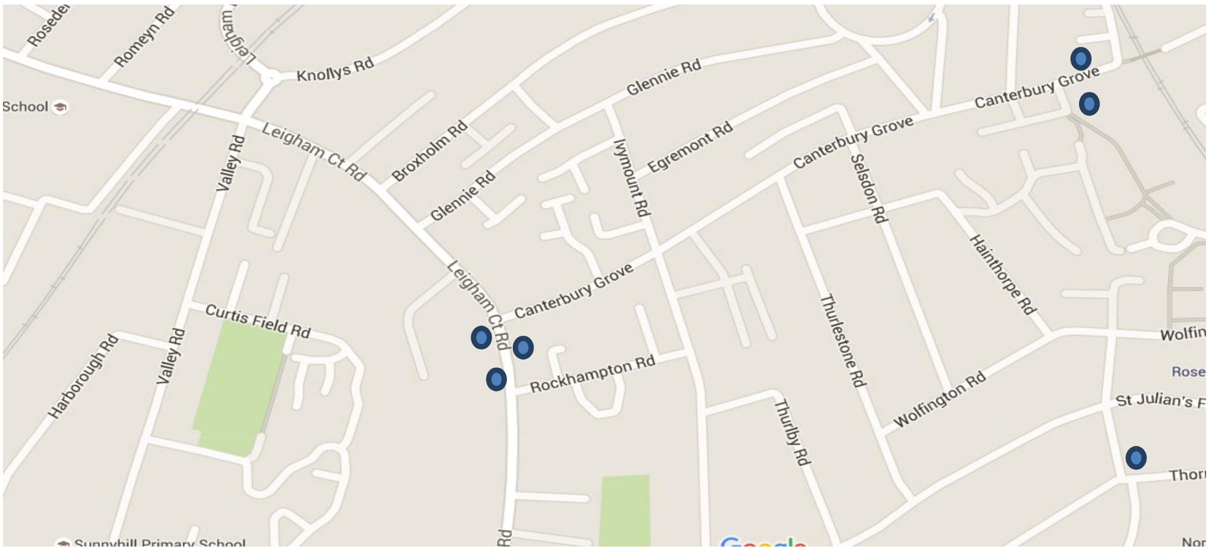

Supplement: Multimedia Appendix 7 [file mhealth_v4i3e106_app7.pdf]
